# Etymologia: Bonferroni Correction

**DOI:** 10.3201/eid2102.ET2102

**Published:** 2015-02

**Keywords:** Bonferroni correction, Emilio Bonferroni, statistical analysis, multiple comparisons

## Bonferroni [bonʹfər-ōʺni] Correction

Named after Italian mathematician Carlo Emilio Bonferroni (1892–1960) but first attributed to Olive Jean Dunn, the Bonferroni correction compensates for multiple comparisons by dividing the significance level by the number of comparisons. The significance level is the probability that a given test will incorrectly find a difference in the sample that is not present in the population (false positive). A significance level of 0.05 is a commonly accepted significance level. If a study tested 5 comparisons, there would be up to a 25% likelihood (0.05 + 0.05 + 0.05 + 0.05 + 0.05) that any one of them would show a significant difference by chance. The Bonferroni correction adjusts for this by dividing the significance level by the number of tests. In this case, the significance level for a given comparison would be 0.01, for an overall risk no larger than 0.05 of falsely detecting a difference.

This technique has been criticized as too conservative, particularly when a large number of tests are used, and it may increase the risk for a false negative. Other tests, such as the Tukey-Kramer and Scheffe method, may reduce this risk.
